# UPLC-MS/MS based diagnostics for epithelial ovarian cancer using fully sialylated C4-binding protein

**DOI:** 10.1002/bmc.4180

**Published:** 2018-01-23

**Authors:** Kazuhiro Tanabe, Koji Matsuo, Masaki Miyazawa, Masaru Hayashi, Masae Ikeda, Masako Shida, Takeshi Hirasawa, Ryuichiro Sho, Mikio Mikami

**Affiliations:** 1 Medical Solution Promotion Department, Medical Solution Segment, LSI Medience Corporation, Tokyo, Japan; 2 Division of Gynecologic Oncology, Department of Obstetrics and Gynecology, Norris Comprehensive Cancer Center, University of Southern California, Los Angeles, California, USA; 3 Department of Obstetrics and Gynecology, Tokai University School of Medicine, Kanagawa, Japan; 4 Department of Obstetrics and Gynecology, Sho Hospital, Tokyo, Japan

**Keywords:** biomarker, clear cell carcinoma, complement 4-binding protein, ovarian cancer, UPLC-MS/MS

## Abstract

Serum levels of fully sialylated C4-binding protein (FS-C4BP) are remarkably elevated in patients with epithelial ovarian cancer (EOC) and can be used as a marker to distinguish ovarian clear cell carcinoma from endometrioma. This study aimed to develop a stable, robust and reliable liquid chromatography–hybrid mass spectrometry (UPLC-MS/MS) based diagnostic method that would generalize FS-C4BP as a clinical EOC biomarker. Glycopeptides derived from 20 μL of trypsin-digested serum glycoprotein were analyzed via UPLC equipped with an electrospray ionization time-of-flight mass spectrometer. This UPLC-MS/MS-based diagnostic method was optimized for FS-C4BP and validated using sera from 119 patients with EOC and 127 women without cancer. A1958 (C4BP peptide with two fully sialylated biantennary glycans) was selected as a marker of FS-C4BP because its level in serum was highest among FS-C4BP family members. Preparation and UPLC-MS/MS were optimized for A1958, and performance and robustness were significantly improved relative to our previous method. An area under the curve analysis of the FS-C4BP index receiver operating characteristic curve revealed that the ratio between A1958 and A1813 (C4BP peptide with two partially sialylated biantennary glycans) reached 85%. A combination of the FS-C4BP index and carbohydrate antigen-125 levels further enhanced the sensitivity and specificity.

## 1 ∣ INTRODUCTION

Epithelial ovarian cancer (EOC) is the most frequent cause of gynecological cancer-related deaths (Siegel, Naishadham, & Jemal, 2013). Early-stage EOC has a promising survival outcome, with a 5-year survival rate of nearly 90% vs the rate of <30% reported for advanced-stage disease (Jacobs & Menon, 2004); accordingly, early EOC detection technologies are urgently needed. Carbohydrate antigen 125 (CA-125) is a standard biomarker for detecting EOC recurrences or monitoring treatment efficacy; however, this marker is compromised by its low specificity, particularly in pre-menopausal women or those with nonmalignant gynecological conditions such as endometrioma (Buamah, 2000) and therefore has not been used for early-stage EOC detection.

We previously identified a C4-binding protein with fully sialylated *N*-glycans (FS-C4BP) as a novel EOC marker (Mikami etal., 2015). FS-C4BP particularly distinguished early-stage ovarian clear cell carcinoma (OCCC) from endometrioma much more reliably than CA-125. A2160, a FS-C4BP peptide with a fully sialylated and fucosylated triantennary glycan and fully sialylated biantennary glycan, exhibited superior sensitivity and specificity as an EOC marker. However, a complicated lectin-based enrichment process is needed to detect A2160, which is present in serum at quite low levels. This prolonged preparation process led not only to a poorer throughput, but also worse accuracy and robustness. Therefore, procedural improvements are necessary if we wish to generalize FS-C4BP as a clinical EOC marker.

AFP-L3, a well-known serum biomarker of hepatocellular carcinoma (Taketa et al., 1993) determined by the ratio between fucosylated (L3) and nonfucosylated (L1 and L2) AFP, has several advantages. First, nonfucosylated glycoforms such as L1 and L2 serve as internal standards that can be used to negate errors generated in the preparation and analysis steps. Second, nontargeted glycoforms cancel the serum protein expression level, thus emphasizing the degree of glycan alteration. Third, a two-glycoform ratio-based diagnosis does not require a standard curve for quantification, and therefore does not require standard molecules. Given the current impossibility of synthesizing pure standard glycoproteins, this advantage was key to the generalization of this method as a universal diagnostic technique. Therefore, we aimed to identify an alternative marker of FS-C4BP with a relatively much higher serum level, compared with A2160, and thus simplify the complicated preparation steps. Next, we aimed to identify a counterpart C4BP glycopeptide for FS-C4BP that could serve as an internal standard. In this study, we used sera from 119 patients with EOC and 127 women without cancer to assess our developed method and compare its performance with that of our previous method.

## 2 ∣ MATERIAL AND METHODS

### 2.1 ∣ Patient samples

A total of 119 serum samples were collected from patients with EOC at the time of ovarian mass detection but prior to the initiation of any treatment. The patients had a mean age ± standard deviation (SD) of 57.5 ± 11.9 years. The noncancer control group (*n* = 127) comprised both healthy women (*n* = 49) and women with gynecological diseases other than ovarian cancer (*n* = 78). Because tests for the current EOC marker, CA-125, often yield false-positive results in menstruating or pregnant women, we collected sera from six pre-menopausal women during their menstrual periods and 43 pregnant women to clarify the advantage of FS-C4BP. The 78 patients with gynecological diseases other than ovarian cancer included 24 cases of endometrioma (EM), 22 cases of uterine fibroid (UF) and 32 cases of ovarian cystoma (CY, Table 1). Although these participants were included because their diseases often cause false CA-125 elevations, the patients in this study were randomly selected and an intentional extraction using CA-125 levels was never performed. Sera of patients with EOC and other gynecological diseases were obtained from Tokai University Hospital (Kanagawa, Japan), and sera of healthy women who were on their menstruation period (ME) and pregnant (PG) were obtained from Sho Hospital (Tokyo, Japan). All blood samples from patients were collected by venous puncture before surgery or any treatment. Serum samples were obtained after centrifugation and stored at −80°C until further examination.

### 2.2 ∣ Study approval

Institutional Review Board approval for the use of patients’ clinical information and serum/tumor samples was obtained at Tokai University (registration number, 09R-082).

### 2.3 ∣ Sample preparation

Ten microliters of a 2 mg/mL aqueous solution of fetal calf fetuin (Sigma, St Louis, MO, USA) was added to each serum sample (20 μL) to check the efficiency of trypsin digestion or the recovery of glycopeptides. Subsequently, trichloroacetic acid in acetone (100 mg/mL, 120 μL; Wako Pure Chemical Industries Ltd, Osaka, Japan) was added to remove serum albumin. After mixing and centrifuging at 13,500 ***g*** for 5 min, the supernatant was removed, and cooled acetone (400 μL) was added to precipitate for washing. The precipitate was centrifuged again at 13,500 ***g*** for 5 min, and the supernatant was removed. Finally, the precipitate was mixed with denaturing solution containing urea (80 μg; Wako Pure Chemical Industries), Tris–HCl buffer (pH 8.5, 100 μL), 0.1 m EDTA solution (10 μL), 1 m Tris (2-carboxyethyl) phosphine hydrochloride (5 μL; Sigma) solution and water (38 μL), and proteins were denatured for 10 min at 37°C. Next, 1 m 2-iodoacetamide (40 μL; Wako Pure Chemical Industries) solution was added to the denaturing solution to protect the thiol residues in proteins. The solution was kept for 10 min at 37°C in the dark, subsequently transferred into a 30 K ultrafiltration tube (Amicon Ultra 0.5 mL; Millipore Corp., Billerica, MA, USA) and centrifuged at 13,500 ***g*** for 30 min to remove denaturing reagents. The denatured proteins trapped on the filter were washed with 0.1 m Tris–HCl buffer (pH 8.5, 400 μL), followed by centrifugation at 13,500 ***g*** for 40 min. Next, 0.1 m Tris–HCl buffer (pH 8.5, 200 μL), 0.1 μg/μL trypsin (20 μL; Wako Pure Chemical Industries) solution and 0.1 μg/μL lysyl endopeptidase (20 μL; Wako Pure Chemical Industries) solution were added to the ultrafiltration tube, and the denatured proteins on the filters were digested for 16 h at 37°C. After digestion, the solution was centrifuged for 30 min at 13,500 ***g***. The filtered solution, which contained digested peptides (including glycopeptides), was transferred to a 10 K ultrafiltration tube (Amicon Ultra 0.5 mL; Millipore Corp.) and centrifuged for 10 min at 13,500 ***g***. Most glycopeptides were trapped on the 10 K ultra-filter, whereas most nonglycosylated peptides were filtered (Tanabe, Kitagawa, Kojima, & Iijima, 2016). The trapped glycopeptide fraction was washed with 10 mm ammonium acetate in 10% (v/v) acetonitrile solution (400 μL), transferred to a 1.5 mL tube, and subjected to drying via vacuum centrifugation. Glycopeptides trapped on the filter were recovered and analyzed by UPLC-MS/MS.

### 2.4 ∣ Liquid chromatography and mass spectrometry

The data of UPLC-MS/MS were acquired on a UPLC system (Agilent HP1200; Agilent Technologies, Palo Alto, CA, USA) equipped with a C_18_ column (Inertsil ODS-4, 2 μm, 100 Å, 100 × 1.5 mm i.d.; GL Science, Tokyo) and coupled with an electrospray ionization quadrupole time-of-flight mass spectrometer (Agilent 6520, Agilent Technologies). Solvent A was 0.1% formic acid, and solvent B comprised 0.1% formic acid in 9.9% water and 90% acetonitrile. Glycopeptides were eluted at 40°C and a flow rate of 0.15 mL/min, using the following gradient program: 0–7 min, 15–30% solvent B; 7–12 min, 30-50% solvent B; and an additional 2 min hold at 100% solvent B. The mass spectrometer was operated in negative mode with a capillary voltage of 4000 V. The nebulizing gas pressure was 30 psi, and the dry gas flow was 8 L/min at 350°C. The injection volume was 5 μL. The precursor ions (*m/z*) and collision energies of A1813 and A1958 were set at 1813.7 and 60 V, and 1958.25 and 60 V, respectively. Extracted ion chromatography data were obtained in the following *m/z* ranges: 1808.4–1810.5 for A1813, and 1885.7–1887.7 for A1958. A1958 and A1813 values were normalized to the corresponding values in serum from a healthy female control.

### 2.5 ∣ Method validation

Two types of validation samples (quality control samples, QC) were prepared. One was QH (high level of FS-C4BP), comprising pooled sera from five patients with EOC, and the other was QL (low level of FS-C4BP), comprising pooled sera from five healthy women. The five dispensed QH and QL samples were treated and analyzed simultaneously within 1 day. The coefficient of variation (CV) of FS-C4BP was assessed to determine intra-reproducibility. These assessments were repeated on three consecutive days, and the CVs (%) of the three averages of five intra-day analyses were evaluated to determine reproducibility. The stability of FS-C4BP was evaluated by comparing data from QC samples at baseline vs a 24 h exposure at room temperature or 4°C. This assessment assumed that the interval from blood sampling to centrifuging, dispensing and refrigeration would depend on individual hospitals. The stability of FS-C4BP after preparation was assessed by comparing prepared QC samples at baseline vs exposure to 4°C for 24 or 48 h. This assessment assumed that the prepared samples (before UPLC-MS/MS analysis) would be left on a UPLC autosampler at 4°C for a maximum of 48 h if more than 100 samples were dealt with. The relationship between the abundance ratio and peak intensity was determined using the following procedure. After QH and QL were mixed at (0, 100%), (20, 80%), (40, 60%), (60, 40%), (80, 20%) and (10, 0%), they were treated and subjected to UPLC-MS/MS; subsequently, target marker intensities were plotted using the abundance ratio, and a correlation coefficient (*R*) of the fitting curve was evaluated. This assessment was performed to evaluate the influence of detector saturation or ion suppression.

### 2.6 ∣ Data analysis

All raw data were analyzed using Masshunter (Agilent Technologies). The area under curve (AUC) of receiver operating characteristic (ROC) analyses and box–whisker plots were performed using Multibase (version 2014; http://www.numericaldynamics.com).

## 3 ∣ RESULTS

### 3.1 ∣ Method development for A1958, a new FS-C4BP target

**FIGURE 1:**
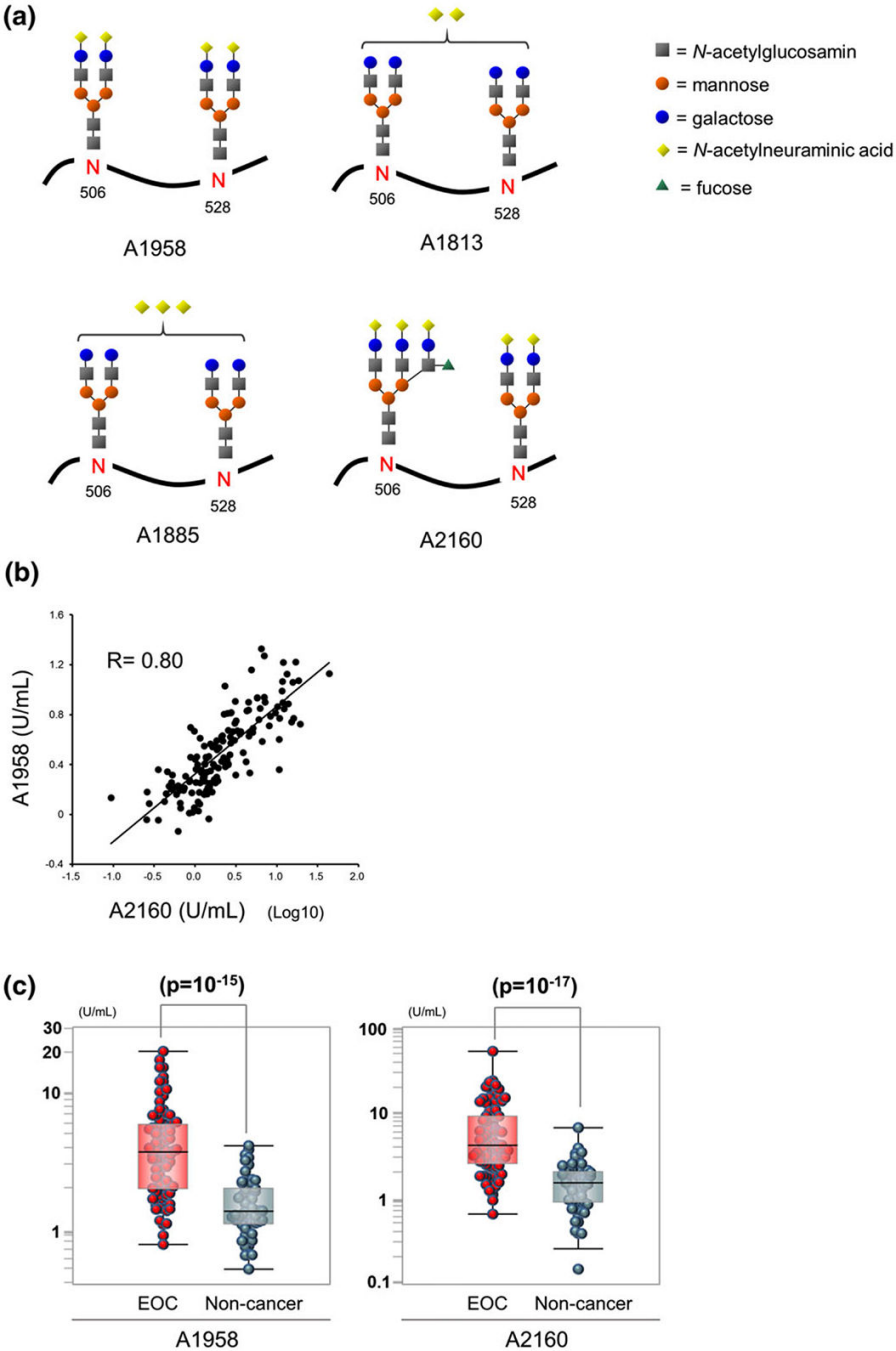
Alpha-chain glycopeptides of C4BP and comparison of A1958 and A2160. (a) Structures of the C4BP alpha-chain glycopeptides, A1958, A2160, A1813 and A1885, that comprise the C4BP alpha-chain DQYVEPENVTIQCDSGYGVVGPQSITCSGNR. (b) Scatter plot of A1958 and A2160 levels in sera from 148 participants. (c) Box-whisker plots of A1958 and A2160 from 90 patients with epithelial ovarian cancer (EOC) and 58 women without cancer

Three major C4BP alpha-chain glycopeptides, A1958, A1885 and A1813, were detected in sera from patients with EOC. A1958 is an FS-C4BP family member with two fully sialylated biantennary glycans, whereas A1885 and A1813 are C4BP peptides containing partially sialylated biantennary glycans and three or two sialic acids, respectively (Figure 1a). All of these glycopeptides were identified by comparing *m/z* and retention time with trypsin digests of a purified human serum C4BP. We discovered that A1958 was expressed in sera at higher concentrations relative to A2160 (Figure 1a, C4BP peptide with a fully sialylated and fucosylated triantennary glycan and a fully sialylated biantennary glycan). The A1958 level correlated well (*R* = 0.80) with the A2160 level (Figure 1b) and was significantly elevated in EOC patient sera (Figure 1c). Although the *p*-value of A1958 (10^−15^) was less than that of A2160 (10^−17^, Figure 1c), the AUC of ROC yielded values as high as 85% in a comparison of 90 patients with EOC and 58 women without cancer women (only comparable samples containing both A1958 and A2160 were used). The UPLC-MS/MS-based A1958 analytical method was optimized as shown in Table 2. Reducing the serum volume from 100 to 20 μL allowed downsizing of the ultra-filtration tube size from 4.0 to 0.5 mL and allowed a higher number of samples per batch (from 16 to 96 samples). The high abundance of A1958 also allowed us to omit the *Aleuria aurantia* lectin (AAL) enrichment step. Given the occasionally unacceptable variability in AAL quality, avoiding this step could not only enhance throughput, but also improve assay robustness. The processes of albumin removal, denaturation, and reductive alkylation were also optimized, and the total preparation step was significantly shortened. The UPLC-MS(/MS) analytical period was also reduced from 60 to 20 min per sample, and mass detection was changed from single MS to MS/MS to increase selectivity.

### 3.2 ∣ The ratio between A1958 with partially sialylated C4BP peptide

**FIGURE 2:**
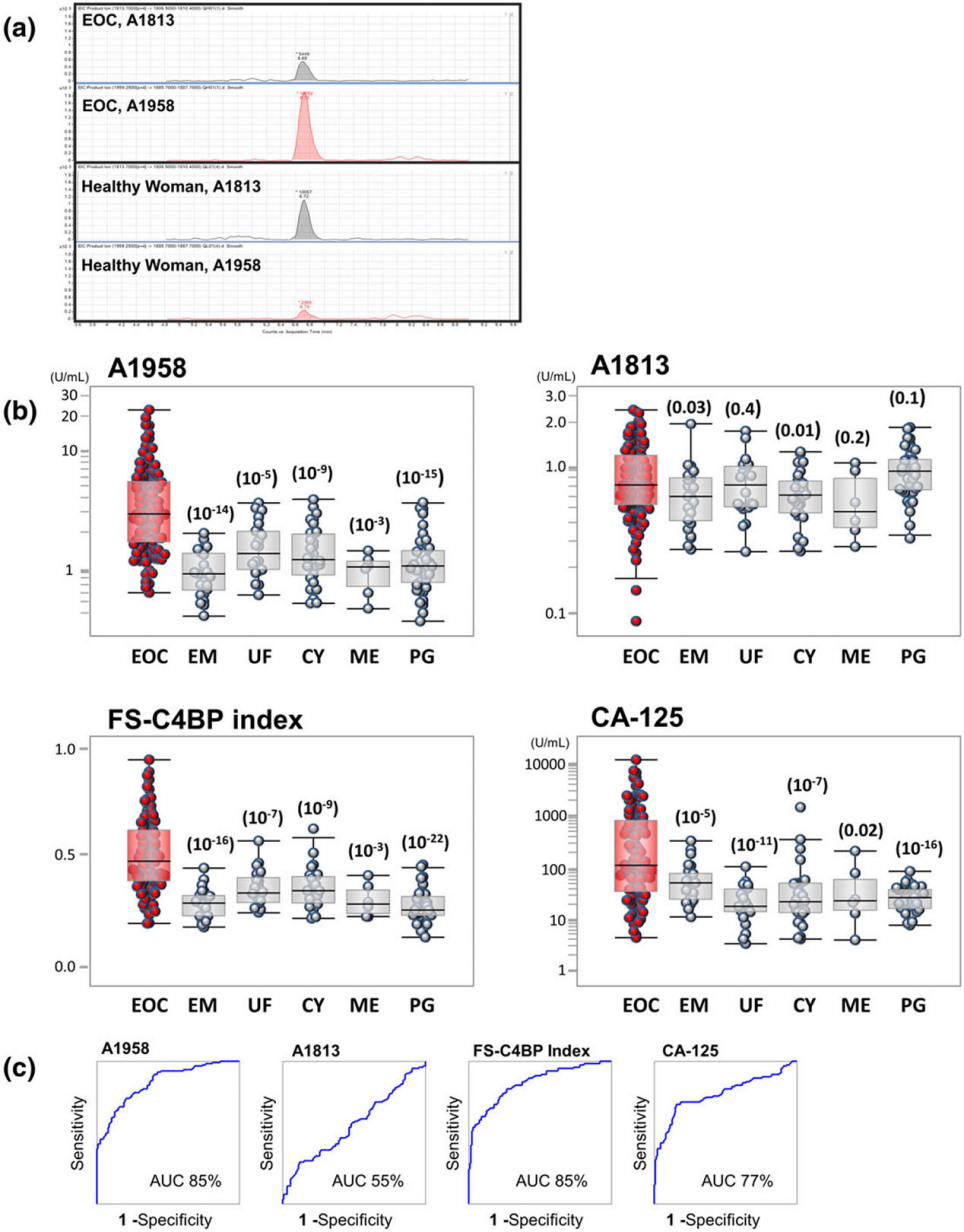
Fully sialylated C4-binding protein (FS-C4BP) index and carbohydrate antigen (CA)-125 levels. (a) Ultra-high-performance liquid chromatography (UPLC)-mass spectrometry (MS)/MS chromatograms of A1813 from EOC serum (upper), A1958 from EOC serum (second), A1813 from healthy control serum (third) and A1958 from healthy control serum (bottom). (b) Box-whisker plots for A1958, A1813, FS-C4BP index and CA-125 for women with EOC, endometrioma (EM), uterine fibroid (UF), benign ovarian cyst (CY), menstruation period (ME) and pregnancy (PG). (c) Receiver operating characteristic curve analyses of A1958, A1813, FS-C4BP index and CA-125 in 119 women with EOC and 127 women without cancer

To provide reliable, accurate A1958 values using mass spectrometry, we would need a counterpart of this C4BP glycopeptide that could correct errors generated during preparation and analysis. We discovered that A1813, a C4BP peptide containing two biantennary glycans with two sialic acids, was an ideal glycoform to serve as an internal standard because it existed at high concentrations in sera and was not affected by EOC development (Figure 2a and b). The AUC of an ROC analysis of A1813 yielded a value of 56% when 119 EOC patients and 127 controls were compared (Figure 2c). The ‘FS-C4BP index’, calculated using the equation FS-C4BP index = A1958/(A1958 + A1813), showed a clearer separation between the EOC patients and controls than did CA-125; furthermore, the AUC (ROC analysis) of the FS-C4BP index (85%) was much higher than that of CA-125 (77%, Figure 2c).

A1885 was not an adequate counterpart for A1958 because it exhibited a slight increase in EOC patients and thus canceled the elevation in A1958 levels when the ratio of these two glycopeptides was calculated.

### 3.3 ∣ Method validation

FS-C4BP index-based diagnostics were validated for reproducibility, stability and the correlation between abundance ratio and peak intensity (Table 3). All results were satisfactory with respect to clinical diagnostics.

### 3.4 ∣ Combination assay with CA-125

**FIGURE 3:**
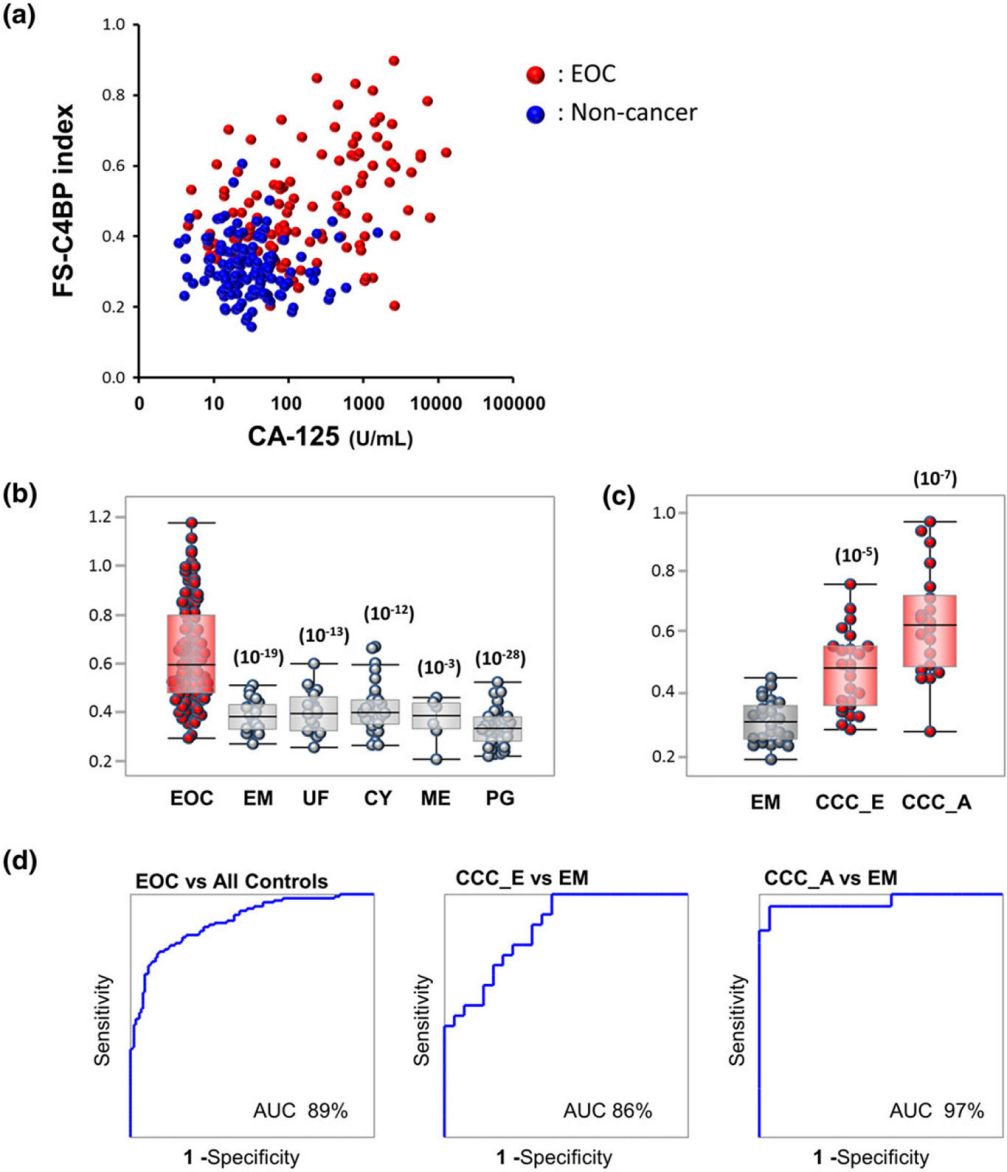
Combination of the fully sialylated C4-binding protein (FS-C4BP) index and carbohydrate antigen (CA)-125. The combination of FS-C4BP and CA-125 was evaluated using an index calculated as follows: combination index = 0.9 × FS-C4BP index + 0.1 × Log_10_(CA-125). (a) Scatter plot of the FS-C4BP index vs CA-125. Black filled circles: patients with EOC; gray diamonds, noncancer controls. (b) Box-whisker plot of the combination index regarding EOC, EM, UF, CY, ME and PG. (c) Box-whisker plots of the combination index for EM, early-stage ovarian clear cell carcinoma (OCCC; CCC_E) and advanced-stage ovarian clear cell carcinoma (CCC_A). (d) Receiver operating characteristic curve analyses of the combination index for EOC vs all controls, OCCC stages I vs endometrioma, and OCCC stage II, III, IV vs endometrioma

Although a slight correlation (*R* = 0.48) was observed between the FS-C4BP index and CA-125, these parameters were almost independent (Figure 3A), indicating that their relationship was complementary. The use of the combination index, calculated as 0.9 × FS-C4BP index + 0.1 × log_10_(CA-125), enhanced the separation between EOC and noncancer patient groups (Figure 3B), and the AUC of the ROC reached 89% when 119 patients with EOC and 127 women without cancer were compared. This combination also indicated a clear separation between patients with endometrioma and those with early-stage (stage I) OCCC (CCC_E; AUC: 86%) and between those with endometrioma and advanced-stage (stages II–IV) OCCC (CCC_A; AUC: 97%, Figure 3c and 3d).

## 4 ∣ DISCUSSION

We aimed to establish a reliable and high-performance diagnostic method for EOC that was based on FS-C4BP, which we previously identified as an EOC marker (Mikami et al., 2015). The simplification of preparation steps and introduction of an MS/MS technique enabled the detection of the C4BP-related glycopeptide, A1958, the diagnostic performance of which was equal to that of A2160 with significantly better throughput.

Compared with immunoassays, MS is advantageous because it can distinguish slight differences in molecular structures. The number of evaluations and utilization of various types of UPLC-MS(/MS) in clinical laboratories have increased (Strathmann & Hoofnagle, 2011). These MS techniques overcome the limitations of conventional immunoassay techniques and provide many advantages such as disease screening (Chace, Kalas, & Naylor, 2003), diagnosis of metabolic disorders (van Scherpenzeel, Steenbergen, Morava, Wevers, & Lefeber, 2015), monitoring of drug therapy (Manicke, Abu-Rabie, Spooner, Ouyang, & Cooks, 2011) and discovery of new biomarkers (Diamandis, 2004). For example, UPLC-MS/MS has become a standard for assays targeting steroid hormones (Stanczyk & Clarke, 2010), vitamin D (Farrell et al., 2012) and thyroid hormones, and has overcome the issues associated with immunoassays (Soldin, Soukhova, Janicic, Jonklaas, & Soldin, 2005). However, challenges remain regarding the clinical application of this technology, including sample preparation, online extraction, throughput, automation, laboratory information system interfacing, inter-instrument standardization, and harmonization and regulation. The key areas in the development of UPLC-MS/MS based diagnostics are simplification of the preparation process and reduction in the UPLC analysis period. However, this analytical period (20 min) is not satisfactory for routine medical test and further improvement, such as increasing flow rate and shortening analytical period, is required.

## 5 ∣ CONCLUSION

In this study, we revealed that A1958, a peptide in the FS-C4BP family containing two fully sialylated biantennary *N*-glycans, correlated well with A2160 and exhibited a novel EOC diagnostic specificity and sensitivity. The FS-C4BP index, defined as the ratio between A1958 and A1813 (a C4BP peptide with partially sialylated biantennary *N*-glycans), increased the robustness and reliability of this method. The validation test of this method satisfied the clinical diagnostic requirements. The combination of the FS-C4BP index and CA125 yielded a superior AUC of ROC, compared with single markers. This combination index could also significantly distinguish Stage I OCCC from endometrioma and is expected to be useful as a monitoring marker for patients with endometrioma.

## Figures and Tables

**TABLE 1:** Summary of patients

Participants			Samples	Age (mean ± SD)
EOC^a^	Serous	26	119	57.5 ±11.9
	Clear cell	44		
	Endometrioid	21		
	Mucinous	14		
	Unclassified	14		
Noncancer	Endometrioma (EM)	24	127	40.1 ± 6.4
	Uterine fibroid (UF)	22		43.6 ± 7.3
	Benign ovarian cyst (CY)	32		53.2 ± 14.4
	Women on menstruation	6		43.7 ± 2.0
	period (ME)			
	Pregnant women^b^ (PG)	43		31.2 ± 4.2

a Stage I/II/III/IV/not determined: 54/8/43/11/3.

b Period of pregnancy, 8/9/10/11/12 weeks: 5/9/13/10/6.

**TABLE 2:** Comparison between previous and improved methods

Method		Previous method	Improved method
FS-C4BP target		A2160	A1958
Preparation	Sample volume	100 μL	20 μL
	Step of removing albumin	90 min	10 min
	Step of denaturing	20 min	10 min
	Step of reductive alkylation	60 min	10 min
	Amount of trypsin	10 μg	2 μg
	Ultrafiltration 30 K	4 mL tube	0.5 mL tube
	Ultrafiltration 10 K	4 mL tube	0.5 mL tube
	Enrichment by AAL	Required	Not required
	Performance	16 samples/batch	96 samples/batch
LC-MS(/MS)	Analytical period	60 min	20 min
	Detection	Single MS	MS/MS

**TABLE 3:** Validation data

		n	RE or CV
Intra-day reproducibility	QL	5	CV 10.4%
	QH	5	CV 1.7%
Inter-day reproducibility	QL	5 × 3 D	CV 11.0%
	QH	5 × 3 D	CV 1.2%
Stability in serum at 25 °C for 24 h	QL	2	RE −3.5%
	QH	2	RE 1.3%
Stability in serum at 4 °C for 24 h	QL	2	RE 4.5%
	QH	2	RE 1.7%
Stability after preparation at 4 °C for 24 h	QL	2	RE 6.9%
	QH	2	RE 1.1%
Stability after preparation at 4 °C for 48 h	QL	2	RE 2.9%
	QH	2	RE 2.1%
Correlation between concentration and peak intensity		5	*R* = 0.99

RE, Relative error (%); QH, high level of FS-C4BP; QL, low level of FS-C4BP; D, days.

## Abbreviations:

AAL: *Aleuria aurantia* lectin
CA-125: carbohydrate antigen 125
CY: ovarian cystoma
EOC: epithelial ovarian cancer
FS-C4BP: fully sialylated C4-binding protein
ME: menstruation
OCCC: ovarian clear cell carcinoma
PG: pregnant
ROC: receiver operating characteristic analyses
UF: uterine fibroid
